# Statistical atlas-based descriptor for an early detection of optic disc abnormalities

**DOI:** 10.1117/1.JMI.5.1.014006

**Published:** 2018-03-06

**Authors:** Fantin Girard, Conrad Kavalec, Farida Cheriet

**Affiliations:** a Polytechnique Montreal, Montreal, Quebec, Canada; b St. Mary’s Hospital, Montreal, Quebec, Canada

**Keywords:** atlases, registration, fundus imaging, retina

## Abstract

Optic disc (OD) appearance in fundus images is one of the clinical indicators considered in the assessment of retinal diseases such as glaucoma. The cup-to-disc ratio (CDR) is the most common clinical measurement used to characterize glaucoma. However, the CDR only evaluates the relative sizes of the cup and the OD via their diameters. We propose to construct an atlas-based shape descriptor (ASD) to statistically characterize the geometric deformations of the OD shape and of the blood vessels’ configuration inside the OD region. A local representation of the OD region is proposed to construct a well-defined statistical atlas using nonlinear registration and statistical analysis of deformation fields. The shape descriptor is defined as being composed of several statistical measures from the atlas. Analysis of the average model and its principal modes of deformation are performed on a healthy population. The components of the ASD show a significant difference between pathological and healthy ODs. We show that the ASD is able to characterize healthy and glaucomatous OD regions. The deviation map extracted from the atlas can be used to assist clinicians in an early detection of deformation abnormalities in the OD region.

## 1 Introduction

**Fig. 1:**
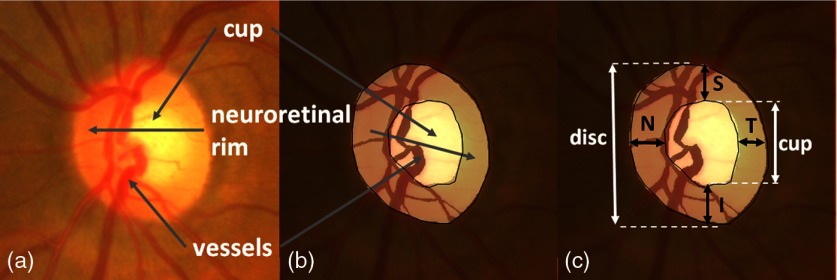
Anatomy of the OD [(a), (b)]; ISNT rule and CDR (c).

Geometric deformation of the optic disc (OD) region is one of the signs of many pathologies affecting the eye. One of the most prevalent pathology that affects the OD is glaucoma. It is characterized by nerve fiber defects caused by an abnormal increase in intraocular pressure.^1^ Glaucoma has a global prevalence of 3.5% in the population aged 40 years and over, the risk increasing with age.^2^ It is the third cause of blindness after diabetic retinopathy and age-related macular degeneration.^3^ Evaluation of OD appearance in the fundus image (see Fig. 1, left/middle) is one of the diagnostic tests used clinically along with visual and intraocular pressure (IOP) tests.^4^ Visual field abnormalities and IOP higher than 22 mm Hg are two clinical findings of glaucoma.^4^ OD appearance can be visualized by standard color fundus photography. Changes can be very subtle, especially in early stages, and clinicians observe different abnormalities in the fundus images to assess early signs of glaucoma. These include generalized or localized enlargement of the optic cup, nerve fiber loss, asymmetry of the cups between the two eyes, narrowing of the neuroretinal rim, and hemorrhages in the OD. The enlargement of the cup is evaluated with respect to the size of the disc. Secondary signs include nasal displacement of the blood vessels, peripapillary atrophy, translucency of the neuroretinal rim, development of vessels overpass, and vessels kinks.^4^ The final diagnosis of glaucoma is supported when at least two or more findings are present, especially in the presence of other risk factors, such as age, sex, family history, identified genes associated with glaucoma, African descent, or myopia.^4^ If detected early, the progression of glaucoma can be slowed down by preventive treatments.^5^

Patients with these symptoms need follow-up exams to check if there is an abnormal progression, but the early signs are difficult to detect on fundus image due to the variability of the OD shapes among patients.

The most commonly used index is the cup-to-disc ratio (CDR), which measures the ratio between the cup diameter and the OD diameter in the vertical direction (VCDR^6^; see Fig. 1). The index can also be defined as the square root of the ratio of the cup area over the disc area called the linear CDR (LCDR).^7^ These CDR measures are used in automatic glaucoma detection and achieve acceptable accuracy.^7^^–^^11^ Using this indicator requires an adequate segmentation of the disc and the cup. Thus, much research effort has been devoted to segmenting the OD and cup in fundus images.^6^^,^^7^^,^^10^^,^^12^^,^^13^ Other indices, such as the ISNT rule (in a normal retina the neuroretinal rim width follows the decreasing order: inferior > superior > nasal > temporal^14^) and the measurement of the nasal displacement of vessels^15^ and vessels kinks,^13^ are used to determine the presence of glaucoma (see Fig. 1, right). While the CDR evaluates general enlargement of the cup, the ISNT rule evaluates its enlargement in the four main directions (temporal, nasal, inferior, and superior).

But the usefulness and accuracy of these indicators are debated among ophthalmologists. Indeed, an OD with a CDR as low as 0.3 can be glaucomatous, while a CDR of 0.8 does not necessarily indicate glaucoma,^16^ which reduces the diagnostic accuracy of this measurement. In a recent study, the ISNT rule, originally assessed on 457 normal fundus in 1988,^17^ was proven to have limited usefulness in assessing glaucomatous damage to the optic nerve.^18^

Simple geometric indicators extracted from fundus images, like the CDR and ISNT rule, are not sufficient to diagnose early geometric abnormalities, which are signs of potential glaucomateous condition of the OD.

The early signs of glaucoma are very subtle, whereas the CDR and ISNT measures describe only advanced stage of glaucoma. Furthermore, the geometric deformations of the vessels, cup, and disc are correlated because their geometric changes occur simultaneously. For example, a large disc size artificially increases the CDR, but it does not indicate pathological condition as nerve fibers need the same space across patients. In addition, when the cup is enlarged, it can induce a nasal rejection of the vessels. The CDR does not evaluate neither vessels deformation nor local deformations of the cup. The ISNT rule does not account for vessels deformation either and evaluates only simple local deformations of the cup.

One way to implicitly exploit all the geometric information contained in the fundus images is to use statistics on deformation fields by computing an atlas of the anatomy of interest. Retinal atlases found in the literature allow detection of abnormalities, such as imaging artifacts^19^ or exudates,^20^ in the fundus image. The registration method used to build the atlas in these methods is an affine registration followed by a thin-plate spline warping guided by the main vessel arches.^19^ Thus, inside the OD, the geometric deformation is not fully described and is limited to affine registration, which restricts the statistical characterization of glaucoma that can be achieved using such an atlas. Finally, other researchers use “topological component analysis”^21^ and “principal orthogonal decomposition”^22^ to characterize the topological changes between one reference exam and its follow-up exams and to quantify the progression of glaucoma. These techniques were not devised to take into account the significant changes between images of different subjects and therefore are not applicable for interpatients statistical analysis.

In this article, we propose to build a statistical atlas of the OD region to analyze the variability of this part of the retina within a healthy population. We build this OD atlas from a population of healthy retinae using the most recent methods of nonlinear registration^23^^,^^24^ and statistical analysis.^25^^,^^26^ One major contribution of this work is a representation of the OD region (cup and rim), including the vessels, resulting in a well-defined atlas. The key to success in constructing our atlas is the use of log-demons registration combined with this local representation of the OD region. The proposed representation captures all the structures including the blood vessels that are likely to deform when the patient is subject to pathological condition. To construct this representation, we reject those vessels that cannot be registered between images and thereby form a probabilistic model containing only the statistically significant vessels. In this way, the atlas construction is not corrupted by the large residuals that would otherwise ensue from keeping all the vessels. An atlas-based shape descriptor (ASD) is then derived from the statistical atlas. This descriptor expresses the shape variability of the whole OD region. The main contribution of this work is to overcome the limitations of simple clinical indicators, such as the CDR by providing a descriptor to identify abnormalities by characterizing any significant deviation from the normal geometric variability. The paper is organized as follows. In Sec. 2, we present the methods for constructing the atlas, including registration, statistical analysis, and defining the ASD. We also introduce a specific representation of the OD region used to build the atlas. In Sec. 3, we present the results of the atlas construction from a healthy population, along with some statistics on the ASD showing its ability to characterize OD normality versus abnormalities. Section 4 concludes this article and presents ongoing and future work.

## 2 Methodology

The construction of the ASD involves five major steps: the extraction of an image patch centered on the OD, the segmentation of the anatomical structures leading to a suitable representation for registration, the construction of an average model through nonlinear registration, the use of statistical tools to represent the atlas variability, and finally the construction of ASD.

**Fig. 2:**
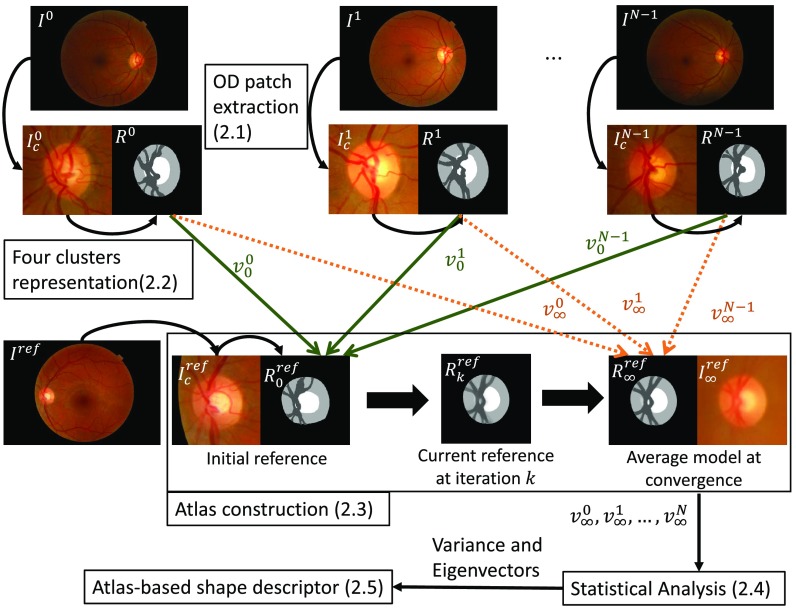
Methodology to form the ASD: each OD is represented as a four-cluster image. The OD region atlas is constructed by iteratively registering the four-cluster representations to the current reference.

Figure 2 illustrates the whole methodology to obtain the ASD, detailed in the following subsections.

### 2.1 Extraction of the Optic Disc Region Patch

In the following, we consider that we have N fundus images noted $I^{i}$ (i for the i’th fundus image) in a healthy population.

#### 2.1.1 Optic disc and macula localization

The method to simultaneously localize the macula and the OD in fundus images presented in Girard et al.^27^ is used. First, several feature maps are processed from the color fundus images, highlighting different properties of the OD or the macula. The OD is defined as a sharp and bright area, radially symmetric and with blood vessels convergence. The macula is defined as an avascular dark area and radially symmetric. Seed points are then placed in the image and evolve toward a set of candidate local minima for the macula. Similarly, circular seeds evolve toward candidate local maxima for the OD. Each seed is given a score calculated from the different maps. Pair scores are formed resulting in the best macula/OD pair noted $\left(x_{\mathrm{OD}}^{i},y_{\mathrm{OD}}^{i}\right)$ and $\left(x_{\mathrm{mac}}^{i},y_{\mathrm{mac}}^{i}\right)$.

#### 2.1.2 Affine registration

Affine transformation occurs inevitably between fundus images from different acquisitions. Translation and rotation are due to the patient placement and eye movement during acquisition. Scaling is due to different ocular sizes in the population under study and also different resolutions from different retina cameras. Finally, a flip is necessary to map right eyes onto left eyes. The centers of the ODs and maculae previously detected are used to parameterize these affine transformations. First, all right eyes are mirrored into left eyes by applying a horizontal flip around the vertical axis passing through the center of the image, whose coordinates are noted $\left(x_{c}^{i},y_{c}^{i}\right)$. If the OD is located to the right of the macula, then the horizontal flip given in Eq. (1) is applied as follows:

$$I^{i}\{x,y)=\{\begin{array}{ll} I^{i}(-x+2x_{c},y), & \mathrm{if}\;(x_{\mathrm{mac}}^{i}-x_{\mathrm{OD}}^{i})<0\\ I^{i}(x,y), & \mathrm{if}\;(x_{\mathrm{mac}}^{i}-x_{\mathrm{OD}}^{i})\geq 0 \end{array}.$$ (1)

Then, a translation and a small rotation due to patient placement and eye movement are parameterized with the OD position $\left(x_{\mathrm{OD}}^{i},y_{\mathrm{OD}}^{i}\right)$ and the angle $\alpha_{i}$ between the horizontal and the line joining the macula and OD centers:

$$\alpha^{i}=\arctan\frac{-y_{\mathrm{mac}}^{i}+y_{\mathrm{OD}}^{i}}{\left(x_{\mathrm{mac}}^{i}-x_{\mathrm{OD}}^{i}\right)}.$$ (2)

The scaling $s=\frac{d^{\mathrm{avg}}}{d^{i}}$ is based on the distance $d^{i}$ between the macula and the OD center:

$$d^{i}=\sqrt{{\left(x_{\mathrm{mac}}^{i}-x_{\mathrm{OD}}^{i}\right)}^{2}+{\left(y_{\mathrm{mac}}^{i}-y_{\mathrm{OD}}^{i}\right)}^{2}}.$$ (3)

We consider that the new image coordinate system is defined by the average of the angles and distances over the set of fundus images. This leads us to define the following affine transformation $A^{i}$ applied to each $I^{i}$:

$$A^{i}|\begin{array}{c} x\\ y \end{array}|=\frac{d^{\text{avg}}}{d^{i}}R_{\left(\alpha^{i}-\alpha^{\mathrm{avg}}\right)}|\begin{array}{c} x\\ y \end{array}|+T^{i}$$ (4)

with

$$R_{\alpha }=|\begin{array}{cc} \cos\;\alpha & -\sin\;\alpha \\ \sin\;\alpha & \cos\;\alpha \end{array}|,$$ (5)

$$T^{i}=|\begin{array}{c} x_{\mathrm{OD}}^{\mathrm{avg}}\\ y_{\mathrm{OD}}^{\mathrm{avg}} \end{array}|-\frac{d^{\mathrm{avg}}}{d^{i}}R_{\left(\alpha^{i}-\alpha^{\mathrm{avg}}\right)}|\begin{array}{c} x_{\mathrm{OD}}^{i}\\ y_{\mathrm{OD}}^{i} \end{array}|,$$ (6)

$$d^{\mathrm{avg}}=\frac{1}{N}\sum_{i}^{N}d^{i},$$ (7)

$$\alpha^{\mathrm{avg}}=\frac{1}{N}\overset{N}{\underset{i}{\sum }}\alpha^{i}\;\text{with}\;\alpha^{i}\in [-\frac{\pi }{2},\frac{\pi }{2}].$$ (8)

After applying the affine transformation, each fundus image is cropped around the OD to form a square patch of size m×m noted $I_{c}^{i}$ that we will use from now on (m is set to 384).

### 2.2 Four-Clusters Representation of the Optic Disc Region

We propose a representation of the OD region that contains the main structures inside that area of the retina: the neuroretinal rim, the cup, and the blood vessels. Generalized or localized enlargement of the optic cup, narrowing of the neuroretinal rim, and nasal displacement of the blood vessels are geometric deformation occurring on these structures for a pathological OD. Thus, the proposed representation takes into account these pathological signs.

#### 2.2.1 Optic disc and cup segmentation

**Fig. 3:**
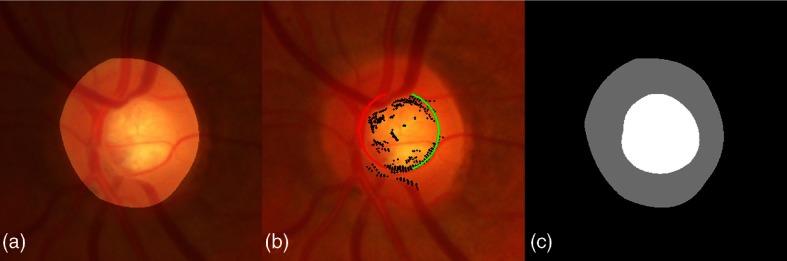
OD and cup segmentation: (a) OD region patch with disc segmentation from Ref. 27; (b) cup segmentation by ellipse fitting (the fitted ellipse is marked in red and green, high polar gradients are marked in black); and (c) cup and neuroretinal rim clusters.

Segmentation of the OD is achieved via a local K-means clustering applied to color coordinates in polar space followed by a polynomial fitting regularization step.^27^ This segmentation method achieves a very competitive overlapping ratio of 0.9 on the MESSIDOR database.^28^ The cup is located using an ellipse fitting method. This is achieved by locating the high gradients in polar space; the resulting ellipse is the one that maximizes gradients along its borders (see Fig. 3).

#### 2.2.2 Vessels segmentation

**Fig. 4:**
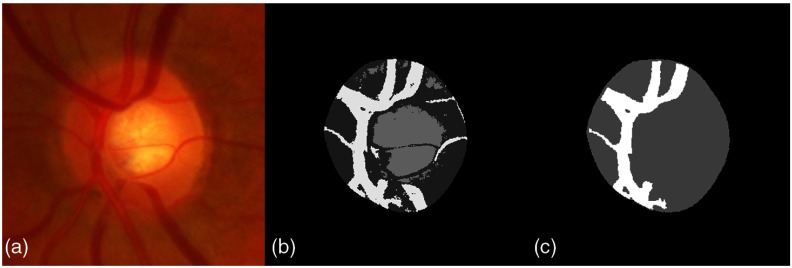
Initial vessels segmentation: (a) OD region patch; (b) K-means clustering with K=3; and (c) selection of the vessels cluster.

Ultimately, we want to be able to analyze not only the optic cup’s deformation but also the vessels’ displacement. One of the signs of pathological OD is the displacement of blood vessels. To segment the vessels inside the OD, a K-means clustering is performed in RGB space, considering that there are three classes inside the OD (see Fig. 4). The class whose average color is closest to dark red is then labeled as the vessels.

**Fig. 5:**
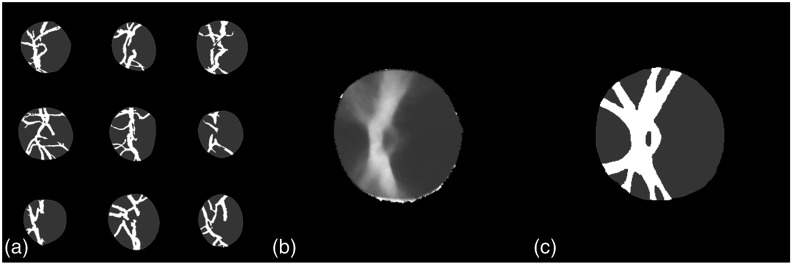
Construction of simplified vessels model: (a) initial vessels segmentations via K-means; (b) vessels average model; and (c) simplified vessels model obtained by thresholding the average model.

However, this initial K-means method segments all the vessels that are visible in the OD region but some smaller vessels will be present in only few of the images. It would not be relevant to consider their displacement in our model. Moreover, due to topological changes that can affect these vessels, it is not guaranteed that we will be able to register them without large residuals between two OD representations, which would corrupt the atlas statistics. Therefore, to find the statistically significant vessels that the registration method will handle correctly, a vessels average model is constructed from the vessels and disc representation obtained previously [see Fig. 4(c)], using the method described in Sec. 2.3.2 below. The resulting vessels average model is used to generate a simplified vessels model that contains only the statistically significant vessels, i.e., those that are common to all the OD images in the atlas population and that we will be able to register without residuals. This simplified vessels model is obtained by thresholding the average model (see Fig. 5), so that at each nonzero locations of the simplified model, the probability to have a vessel after registration onto the vessels average model is over 75%. This value is a trade-off that is set to have the most simple vessels model while keeping the most significant vessels.

**Fig. 6:**
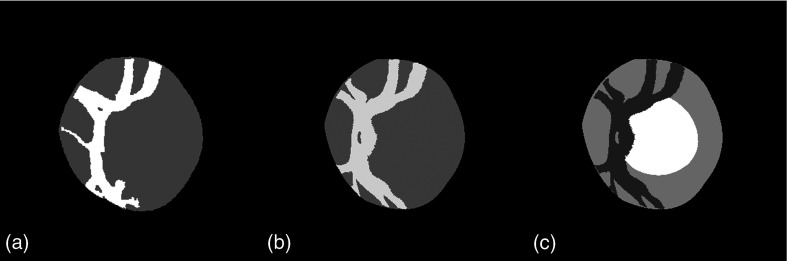
Construction of four-cluster representation: (a) initial vessels segmentation via K-means; (b) simplified vessels model registered onto K-means vessels segmentation; and (c) final four-cluster representation.

We can observe in Fig. 5 that this simplified model is actually related to the main branches of the vessels in the OD. The final vessel segmentation is obtained by registering this simplified vessel model onto the initial K-means vessel segmentation using the log-demons algorithm,^23^ presented in Sec. 2.3.1 below [see Fig. 6(b)]. We end up with an image comprising four clusters representing the background, the vessels, the cup, and the rim [Fig. 6(c)]. The OD region atlas, constructed from this simplified four-clusters representation, is noted R.

### 2.3 Construction of the Optic Disc Region Atlas

The atlas construction method consists in finding the average model and the pairwise geometric transformations between the average model and each OD image.

#### 2.3.1 Optic disc region registration

The log-demons algorithm^23^ is used to perform the pairwise registration between two OD region representations $R^{i}$ and $R^{\mathrm{ref}}$. The registration is modeled by a deformation field that encodes at each image position the displacement vector. The log-demons algorithm is a fast iterative method that enforces the diffeomorphic properties of the resulting deformation field by computing stationary velocity fields defined by the following ordinary differential equation $v(x)=\frac{d}{dt}\phi (t)$. The exponential of the velocity field is a solution of this ordinary differential equation at time 1, i.e., ϕ=exp(v). The diffeomorphic properties of the transformation ensure that no folding will appear in the deformation field. This is the model we want for the OD region because the geometric deformation that appears in this region should be smooth and with no folding. The fact that we manipulate velocity fields in the exponential map guarantees that the corresponding deformation field ϕ is diffeomorphic. The log-demons algorithm can be formulated as a minimization of energy E with the resulting velocity field v and an auxiliary variable $v_{c}$, called the correspondences. This variable is introduced to separate the energy minimization into two steps: one with respect to the correspondences $v_{c}$ with v fixed and the other with respect to the transformation v with $v_{c}$ fixed:

$$E(R^{i},R^{\mathrm{ref}},v,v_{c})=\frac{1}{\sigma_{i}^{2}}{\left\|R^{\mathrm{ref}}-R^{i}\circ \;\exp(v_{c})\right\|}^{2}+\frac{1}{\sigma_{x}^{2}}{\left\|v_{c}-v\right\|}^{2}+\frac{1}{\sigma_{T}^{2}}{\left\|\nabla v\right\|}^{2},$$ (9)

where $\sigma_{i}^{2}$ is the error we allow on the intensities, $\sigma_{x}^{2}$ is the error we allow between the transformation and the correspondences, and $\sigma_{T}^{2}$ is a smoothing parameter. At each iteration of the log-demons algorithm, we first find the update u to apply to the velocity field, which is the minimization of the first part of Eq. (9) (we make the same assumptions as in Vercauteren et al.,^23^ namely that $\sigma_{x}^{2}\approx 1$ and $\sigma_{i}^{2}\approx \|R^{\mathrm{ref}}-R^{i}\circ \exp(v){\|}^{2}$):

$$u=\nabla \overset{˜}{R^{i}}\frac{R^{\mathrm{ref}}-\overset{˜}{R^{i}}}{{\left\|R^{\mathrm{ref}}-\overset{˜}{R^{i}}\right\|}^{2}+{\left\|\nabla \overset{˜}{R^{i}}\right\|}^{2}},$$ (10)

where

$$\overset{˜}{R^{i}}=R^{i}\circ \exp(v).$$ (11)

Then, the second minimization is performed with two Gaussian regularizations corresponding to convolutions with Gaussian kernels: one, which has a fluid behavior, on the update field obtained in Eq. (10); the other, which corresponds to an elastic regularization, on the velocity field itself:

$$v=v+u*K_{\mathrm{fluid}},$$ (12)

$$v=v*K_{\mathrm{diff}},$$ (13)

where $K_{\mathrm{fluid}}$ and $K_{\mathrm{diff}}$ are Gaussian kernels with standard deviations of $\sigma_{\mathrm{fluid}}$ and $\sigma_{\mathrm{diff}}$, respectively.

The alternation between the two minimization steps (correspondences update and field regularization) accelerates the minimization of the energy. The addition in Eq. (12) is possible due to the Baker–Campbell–Hausdorff approximation that simplifies the addition of the update u to the velocity field [exp(v)∘exp(u)≈exp(v+u)]. The scaling and squaring method^29^ is used to efficiently calculate exponentials of fields. A multiresolution scheme is used to accelerate the computation by reducing the number of iterations of the minimization at each scale. We first start with a downscaled representation and apply the resulting velocity field to the upscaled representation.

#### 2.3.2 Average model construction

The average model is constructed following the iterative method of Guimond et al.^24^ A reference $R_{\left(k=0\right)}^{\mathrm{ref}}$ is first arbitrarily chosen from the set of OD images. At each iteration k, each OD representation $R^{i}$ is registered to $R_{k}^{\mathrm{ref}}$ by means of the diffeomorphic transformation $\phi_{k}^{i}=\exp(v_{k}^{i})$ obtained with the log-demons algorithm.

After registering each $R^{i}$ to $R_{k}^{\mathrm{ref}}$ with the corresponding velocity field $v_{k}^{i}$, we average the intensities of the resulting registered images:

$$R_{k}^{\mathrm{re}f^{\prime }}=\frac{1}{N}\overset{N-1}{\underset{i=0}{\sum }}R^{i}\circ \exp(v_{k}^{i}).$$ (14)

The inverse of the average velocity field is next applied to $R_{k}^{\mathrm{re}f^{\prime }}$. This transformation brings the current reference toward the average model. As the log-demons method returns a velocity field, it can be directly averaged or inverted using the log-Euclidean framework described in Arsigny et al.,^25^ which simplifies calculation of the transformation to apply to $R_{k}^{\mathrm{re}f^{\prime }}$:

$$R_{k+1}^{\mathrm{ref}}=R_{k}^{\mathrm{re}f^{\prime }}\circ \exp(-\frac{1}{N}\overset{N-1}{\underset{i=0}{\sum }}v_{k}^{i}).$$ (15)

The final average model $R_{\infty }^{\mathrm{ref}}$ is obtained when the inverse of the average velocity field approaches a null displacement field, i.e., $R_{\infty }^{\mathrm{ref}}\circ \exp(-\frac{1}{N}\overset{N-1}{\underset{i=0}{\sum }}v_{\infty }^{i})=R_{\infty }^{\mathrm{ref}}$. A few iterations (less than 10) are usually sufficient to reach this solution.

The RGB average model $I_{\infty }^{\mathrm{ref}}$ can be calculated by applying the final velocity fields $v_{\infty }^{i}$ to the three-color channels:

$$I_{\infty }^{\mathrm{ref}}=\overset{N-1}{\underset{i=0}{\sum }}I_{c}^{i}\circ \exp(v_{\infty }^{i}).$$ (16)

### 2.4 Statistical Analysis

The statistical analysis step aims at studying the variability of geometric deformations in the atlas population. We have constructed the average model that allows us to compute the velocity fields $v_{\infty }^{i}$ from all the images of the atlas onto the average model. These velocity fields represent the range of geometric deformations that exists in the set of OD regions used to build the atlas. Since the space of velocity fields is a vector space (i.e., the addition of two velocity fields is meaningful), the standard statistical analysis tools are available.

#### 2.4.1 Variance field

First, we calculate the variance of the velocity fields. The variance field can be formulated as follows:

$$\sigma_{R_{\infty }}^{2}=\frac{1}{N}\sum_{i=0}^{N-1}v_{i}^{2}.$$ (17)

The variance field quantifies the expected velocity (or deformation) at each position, thereby revealing the locations in the OD region having the highest geometric variability in the atlas population.

#### 2.4.2 Principal component analysis

Another way to study the variability more globally is to identify the principal modes of variation using principal component analysis (PCA). Each velocity field contains $M=2m^{2}$ velocities at each point of the grid of size m×m. If we want to construct the M×N matrix V of all velocity fields from the N images, $V=(v^{0},v^{1},\cdots ,v^{N-1})$ the covariance matrix $\Sigma =VV^{T}$ will be huge and the Eigen decomposition $\Sigma =P\Lambda P^{T}$, with P the matrix of eigenvectors, exceedingly costly to compute. Furthermore, the decomposition will lead to (N−1) meaningful eigenvectors as the covariance matrix will be of rank (N−1). Turk and Pentland^30^ showed that VE corresponds to the eigenvectors matrix P of the covariance matrix, where E is the eigenvector matrix of the N×N matrix $\Gamma =V^{T}V$.

We will use these two atlas statistics, namely the variance field and PCA, to propose the ASD.

### 2.5 Atlas-Based Shape Descriptor

Let us consider a new fundus image $I^{\mathrm{new}}$. First, we perform macula and OD localization and apply the affine registration to obtain the OD region patch $I_{c}^{\mathrm{new}}$. Second, we compute the corresponding four-clusters representation $R^{\mathrm{new}}$, as shown in Sec. 2.2 above. Third, we project $R^{\mathrm{new}}$ onto the atlas by computing its velocity field $v^{\mathrm{new}}$ to the average model $R_{\infty }^{\mathrm{ref}}$. The local deviation map $d^{\mathrm{new}}$ is computed by dividing the velocity field by the variance field:

$$d^{\mathrm{new}}=\frac{v^{\mathrm{new}}}{\sigma_{R_{\infty }}}.$$ (18)

Local velocity values are thereby weighted by the corresponding variance values: where the atlas variance is high, a high velocity will be less significant; on the contrary, where the atlas variance is low, a low velocity will become more significant.

The projection $p_{j}$ of the new velocity field $v^{\mathrm{new}}$ onto the j’th mode of variation (from the PCA), can be written as follows:

$$p_{j}=v^{\mathrm{new}}\cdot {\left(VE\right)}_{j}.$$ (19)

The ASD we propose is composed of the maximum $d_{\max}$ and the mean $d_{\mathrm{avg}}$ of the local deviation map and of the projections onto all the modes of variation:

$$\mathrm{ASD}=[d_{\mathrm{avg}},d_{\max},p_{0},p_{1},\cdots ,p_{N-1}].$$ (20)

## 3 Experiments and Discussion

We first validated the construction of the statistical atlas by comparing the registration residuals resulting from using different representations and different registration methods. Then, the OD statistical atlas was further validated by analyzing the average model and its variance field and principal variation modes. Finally, we examined whether the ASD was able to distinguish between healthy cases and pathological conditions, such as glaucomateous or prone to develop abnormalities ODs.

### 3.1 Construction of the Optic Disc Atlas

We selected 60 healthy fundus images from the MESSIDOR database,^28^ each having a wholly visible OD and cup and therefore a high confidence in the segmentation accuracy, to construct the statistical atlas. These images were captured using a Topcon TRC NW6 nonmydriatic retinal camera with a 45-deg field of view. The image sizes were 1440×960,2240×1488 or 2304×1536  pixels.

A glaucoma specialist was asked to eventually correct the automatic results of the segmentation by manually adjusting the optic cup and disc boundaries. This step was required to avoid any bias in the atlas construction method and the proposed descriptor.

To validate the construction of the OD atlas, including the choice of representation and the registration method, we quantitatively evaluated the residuals from the registration phase. The residual is defined as follows, with $R_{\infty }^{\mathrm{ref}}$ the average model:

$${\text{Residual}}^{i}={\left\|R_{\infty }^{\mathrm{ref}}-R^{i}\circ \exp(v^{i})\right\|}^{2}.$$ (21)

The regularization parameters of the log-demons algorithm, $\sigma_{\mathrm{fluid}}$ and $\sigma_{\mathrm{diff}}$, were both set to 1.5 to constrain the smoothness of the transformation. These values were set by minimizing the registration residuals on a random set of OD representation from MESSIDOR dataset.

We compare the residuals from the atlas construction to evaluate the contribution of the log-demons registration method over simpler affine registration used in this work and in Lee et al.^19^ Likewise, the proposed four-clusters representation of the OD region was compared to the grayscale representation, obtained by averaging the R,G, and B intensities. The impact of a large registration residual is that when we average the registered image intensities to form the average model, the edges will be blurry and hence not well preserved. Furthermore, the variability will not be correctly calculated as the ODs will not be registered correctly onto the average model.

**Fig. 7:**
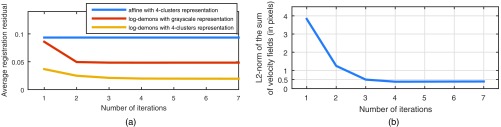
(a) Registration residual during average model construction for different registration methods and OD region representations. (b) Norm of the average of velocity fields $\left\|\frac{1}{N}\sum_{i=0}^{N}v_{k}^{i}\right\|$ during average model construction.

Figure 7 shows how the residuals evolve as a function of the number of iterations in the average model construction process. Clearly, using the four-cluster representation is better than the grayscale representation and log-demons registration performs better than affine registration. We achieve the best average residual using log-demons and the four-cluster representation. As expected, the affine registration residual does not decrease after the first iteration because the average model is directly calculated from the affine transformation. From these results, we can first see that the smooth diffeomorphic transformation, which has more degrees of freedom than an affine transformation, and reduces registration errors. Second, the four-cluster representation is less complex to register for the log-demons than the grayscale representation. The grayscale representation leads to incorrect registrations because the gradients and the disc and cup edges that contribute to the velocity update are noisy and the small vessels are difficult to register. The strength of our representation is that we extract only the statistically significant vessels inside the OD region and the registration is not corrupted by vessels that are not present in the whole population. For example, the cilioretinal artery coming from the temporal side of the OD is present only in 30% of retinae.^1^

We evaluate that the resulting average model is well defined by calculating the norm of the average of velocity fields $\left\|\frac{1}{N}\sum_{i=0}^{N}v_{k}^{i}\right\|$ and observe how this quantity evolves throughout the minimization (see Fig. 7). The norm of the average of velocity fields converges after four iterations and its final value corresponds to a change of less than 0.5 pixels on average.

**Fig. 8:**
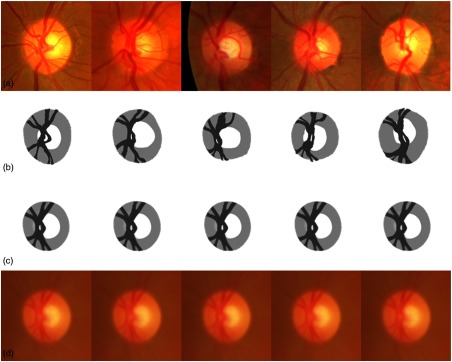
Influence of initial reference selection: (a) initial image chosen as reference; (b) corresponding four-cluster representation $R_{k=0}^{\mathrm{ref}}$; (c) resulting final average model $R_{\infty }^{\mathrm{ref}}$; and (d) corresponding average model $I_{\infty }^{\mathrm{ref}}$ in RGB space.

**Fig. 9:**
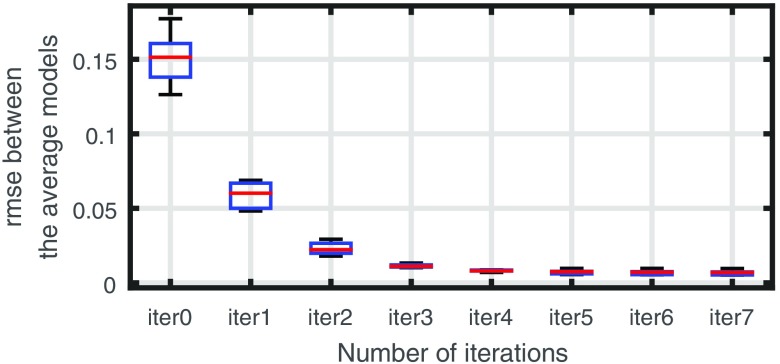
Root mean square error between the five average models constructed from five different initial reference images.

Next, we conducted a test to determine the influence of the choice of initial reference image $R_{k=0}^{\mathrm{ref}}$; five different images, chosen randomly, were tested. We can observe, in Fig. 8, the initial references, their four-cluster representations, and the corresponding final average models. The resulting average models are very similar. This is confirmed by the fact that after four iterations, the average and standard deviation of the root-mean square error of intensities between the five average models is very low (see Fig. 9).

These results indicate that the average model construction behaves well with the representation we have chosen and that it is robust with respect to the choice of the first reference image.

### 3.2 Optic Disc Atlas Analysis

**Fig. 10:**
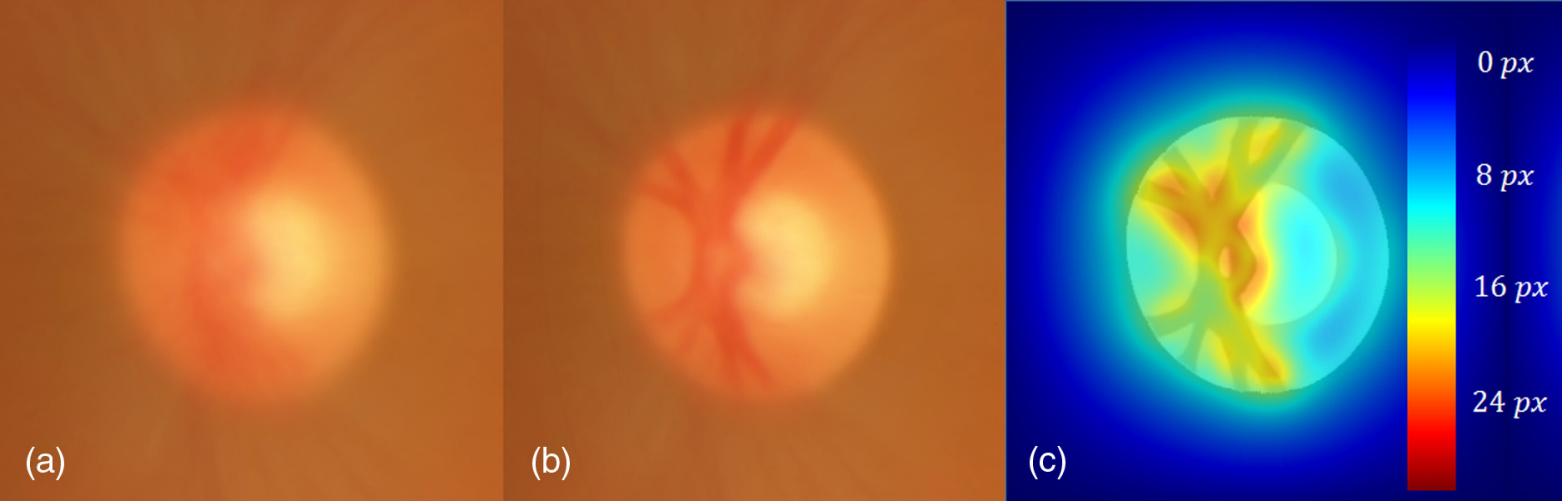
Atlas analysis: (a) average model calculated using only affine registration; (b) average model using log-demons algorithm; and (c) variance map of model in (b).

We can now present the results of the statistical analysis of the average model and its variability measures (local variance and principal components). First, we analyzed the average model qualitatively. In Fig. 10, we can observe that the average model constructed with only affine registration is very blurry. By contrast, using the log-demons registration results in well-preserved edges of the principal anatomical structures, namely the cup, the disc, and the vessels, while keeping a smooth and invertible transformation. The variance field is shown in Fig. 10(c). This map emphasizes that the variability is high for the vessels inside the OD, whereas it is moderate on the boundaries of the OD and cup and low inside the neuroretinal rim.

**Fig. 11:**
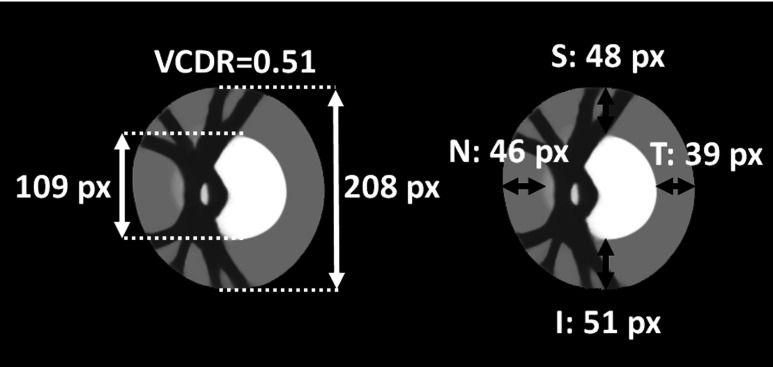
Anatomical validity of average model: VCDR measure and conformity with ISNT rule (lengths indicated in pixels.)

The average OD model is slightly elliptical with the vertical axis as the major axis, which is consistent with normal anatomy.^4^ The LCDR of the average model is 0.49 and the VCDR is 0.51, which are close to the average of the CDR measures in the atlas population (0.51 for both the LCDR and VCDR). The cup’s location is slightly temporal with respect to the center of the OD and the ISNT rule (I>S>N>T) is respected (see Fig. 11).

The PCA applied to the OD atlas is another way to visualize the variability. Rather than expressing the variability locally, the eigenvectors represent velocity fields and therefore global displacement. Each eigenvector explains a proportion of the total variability equal to its eigenvalue divided by the sum of all eigenvalues.

**Fig. 12:**
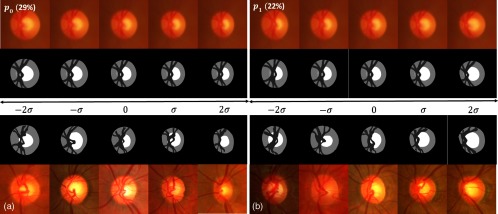
First (a) and second (b) mode of variation: (first row) $\exp({\left(VE\right)}_{0})$ applied to RGB average model; (second row) corresponding four-clusters average model; (last two rows) closest element of the database to the corresponding $\exp(\alpha {\left(VE\right)}_{1,2})$, with α∈[−2;−1;0;1;2].

We observe the first eigenvector, explaining 29% of the variability, in Fig. 12. This figure shows the principal deformation covering the range of variability from −2σ to 2σ and the corresponding closest OD image from the atlas projected onto the eigenvector. The first mode corresponds to a simultaneous enlargement of the disc and the cup. The size of the cup is more variable horizontally, thus a vertical enlargement of the cup would more likely be abnormal than a horizontal one.

The second eigenvector, explaining 22% of the variability, corresponds to a nasal displacement of the vessels as they are pushed by the enlargement of the cup (see Fig. 12 left). Interestingly, this mode of variation characterizes a type of deformation that is one of the less-specific clinical signs of glaucoma.^4^

**Fig. 13:**
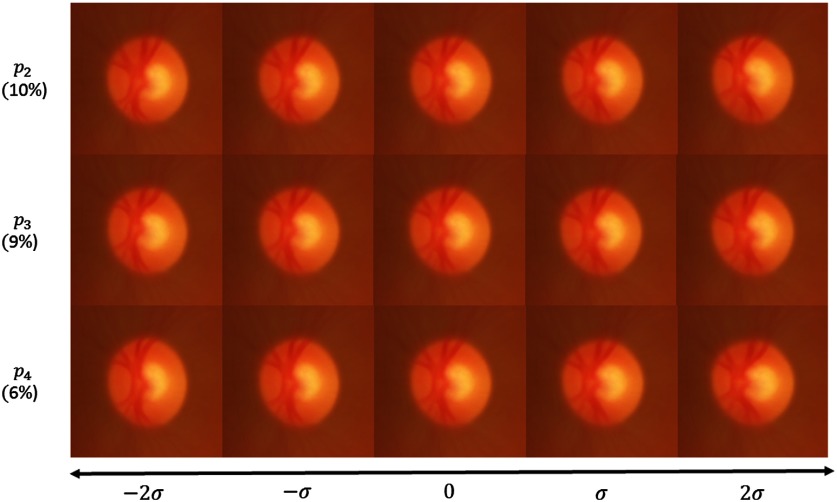
Third, fourth, and fifth modes of variation applied to RGB average model [$\exp({\left(VE\right)}_{i})$] with i∈[2;3;4]).

The two following eigenvectors, explaining, respectively, 10% and 9% of the variability, are close to small rotations with different centers (see Fig. 13). These two eigenvectors are certainly related to the eye placement during the image acquisition. The fifth eigenvector (accounting for 6% of the variability) is a vertical stretching of the OD combined with a vertical stretching of the cup (see Fig. 13). These first five eigenvectors explain 76% of the variability in the atlas population.

**Fig. 14:**
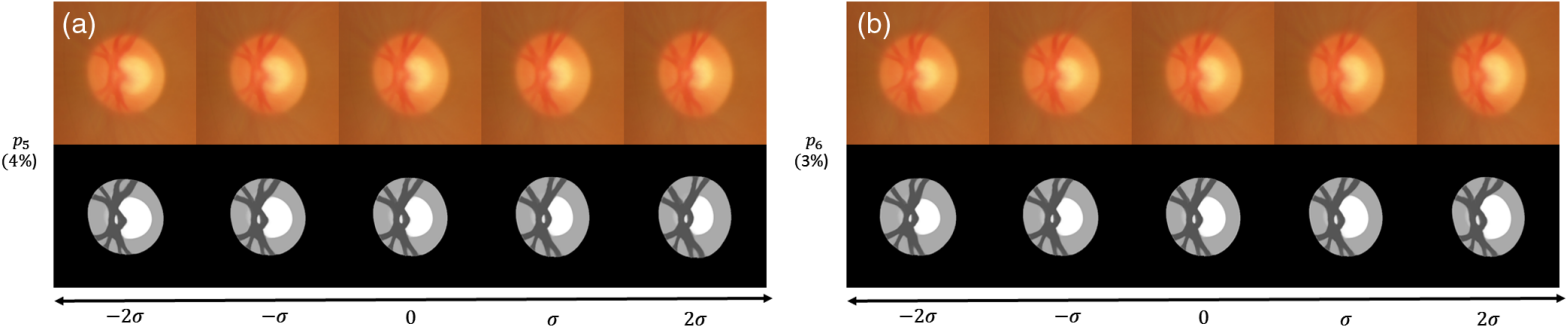
(a) Seventh mode of variation applied to RGB and four-clusters average model $\left[\exp({\left(VE\right)}_{6})\right]$. (b) Sixth mode of variation applied to RGB and four-clusters average model $\left[\exp({\left(VE\right)}_{5})\right]$.

The sixth eigenvector, shown in Fig. 14, explains 4% of the variability and corresponds to a horizontal enlargement of the cup while the disc boundary does not move. The seventh eigenvector, illustrated in Fig. 14, explains 3% of the variability and corresponds mainly to a vertical enlargement of the cup. It is important to note that these two modes characterize specific deformations that can help to detect glaucoma.

**Fig. 15:**
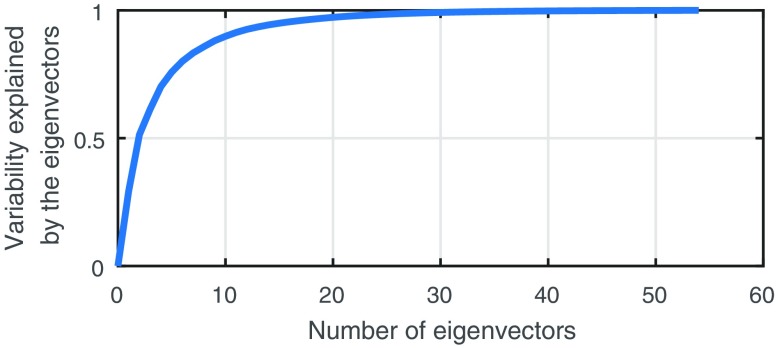
Cumulative percentage of variability explained by the eigenvectors.

The first 10 eigenvectors explain 90% of the variability, whereas the first 20 account for 96.4% of the variability (see Fig. 15).

### 3.3 Assessing Abnormal Conditions Using the Atlas-Based Shape Descriptor

We want to evaluate the ability of the ASD to capture early changes in the geometry of OD and characterize pathological cases. Experiments are conducted on two datasets. The first one contains 16 OD-centered fundus images from Ibn-El-Haythem, Algers, Algeria. These fundus images have been identified as pathological by two ophthalmologists. Pathological case present symptomatic signs and needs, according to the opthalmologists, close follow-up examination to control the potential evolution of the pathology, which is important to detect as early as possible. For the second dataset, 255 fundus images were acquired from the CARA screening platform (Diagnos Inc., Montreal, Canada). These images were captured using digital retina cameras from different manufacturers (Centervue, Canon, Topcon and Zeiss) with a 45-deg field of view and are of size 1620×1444, 2196×1958, 2592×1944, or 3872×2592  pixels. Among the 255 images, 126 fundus images were identified as glaucomatous cases by two ophthalmologists. The other 129 fundus images were not suspected to have glaucoma and considered to be healthy.

**Fig. 16:**
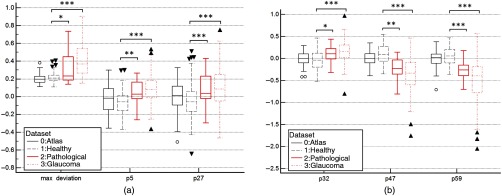
(a) Distribution of measures between the four image sets, for $p_{32}$, $p_{47}$, and $p_{59}$. P-value of Mann–Whitney test of ***, <0.0001; **, <0.001; *, <0.01. (b) Distribution of measures between the four image sets, for $d_{\max}$, $p_{5}$, and $p_{27}$. P-value of Mann–Whitney test of ***, <0.0001; **, <0.001; *, <0.01.

The box plots of the distribution of several ASD components are shown in Fig. 16 for the four resulting populations (atlas, healthy, pathological, and glaucomateous). We can see that for all of these measures, the atlas and healthy groups’ distributions follow each other closely, whereas the pathological and glaucomateous group is always shifted with respect to the other two. The results of Mann–Whitney test show strong difference between healthy and pathological or glaucoma population with most of the time a p-value less than 0.0001 (see Table 1).

**Table 1:** Significance difference between healthy and abnormal OD with CDR and ASD.

	Healthy	Pathological		Glaucoma	
	Mean±Std	Mean±Std	p-value^a^	Mean±Std	p-value^a^
LCDR	0.48±0.05	0.54±0.05	<0.0001	0.59±0.07	<0.0001
Max deviation	0.22±0.05	0.31±0.17	<0.01	0.41±0.19	<0.0001
$p_{5}$	−0.07±0.12	0.05±0.13	<0.001	0.08±0.16	<0.0001
$p_{27}$	−0.06±0.20	0.09±0.18	<0.0001	0.11±0.23	<0.0001
$p_{32}$	−0.05±0.22	0.09±0.20	<0.01	0.17±0.24	<0.0001
$p_{47}$	0.10±0.09	−0.23±0.24	<0.001	−0.37±0.38	<0.0001
$p_{59}$	0.05±0.19	−0.28±0.21	<0.0001	−0.49±0.47	<0.0001

a For Mann–Whitney test.

**Fig. 17:**
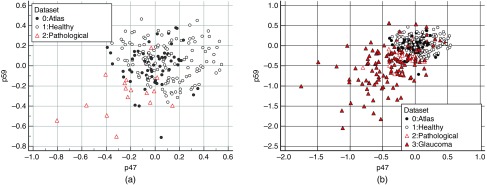
(a) Scatterplot of atlas, healthy, and pathological along two components of the ASD. (b) Scatterplot of atlas, healthy, pathological, and glaucomateous along two components of the ASD.

Scatter plot on the two variables $p_{47}$ and $p_{59}$ show that these two components of the ASD are uncorrelated (see Fig. 17).

**Fig. 18:**
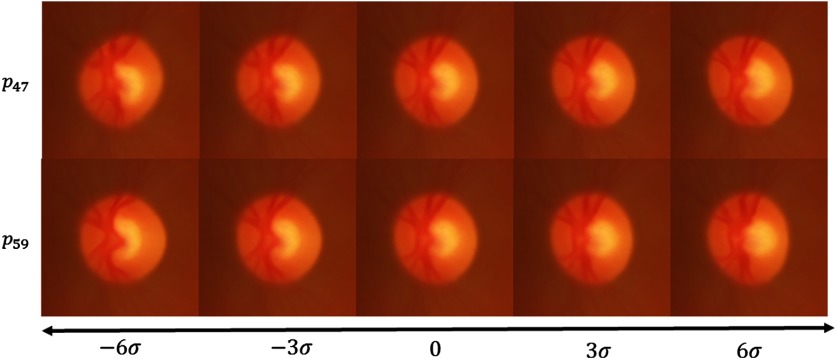
$p_{47}$ and $p_{59}$ corresponding mode of variation applied to the RGB average model.

These components of the ASD correspond to the projection on a specific eigenvector of the atlas. We have already seen that $p_{5}$ looks like an enlargement of the cup horizontally. In Fig. 18, we show the projection corresponding to $p_{47}$ and $p_{59}$. Interestingly, this seems visually to correspond to a vertical and horizontal displacement of the cup, respectively.

**Fig. 19:**
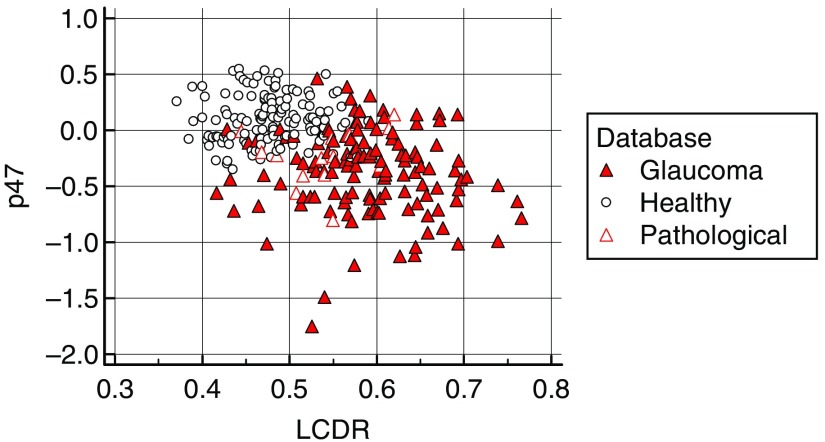
Scatterplot showing that CDR is uncorrelated with the measure extracted from the ASD.

We can observe in Fig. 19 that the LCDR is uncorrelated with $p_{47}$. The LCDR is the square root of the ratio of the cup area over the disc area. It shows that the ASD has at least one component with discriminative information not present in LCDR.

In summary, several components of the ASD show significant differences between glaucomatous and healthy ODs while the healthy ODs lie inside the expected variability of the atlas. While some components of the ASD have low discriminating power to characterize glaucoma by themselves, most of the components of the ASD are uncorrelated with each other, which is very promising for the prospect of combining several ASD components to improve their discriminating power and specialize them for a specific pathology affected by specific geometric deformations.

## 4 Discussion and Conclusion

We have proposed a method to construct a statistical ASD to represent the geometric deformation of the OD region in fundus images. It is the first time that a validated atlas of the OD region is constructed. The atlas construction methods have been validated, and the resulting components of the ASD have been analyzed. Finally, we have showed that these components were able to characterize discriminative deformation and therefore distinguish between healthy ODs and pathological ODs.

The CDR is a good measure for comparative exam but is not able to deal with the variability of OD shape among patients, for example, OD size. This is the strength of our new atlas approach that through the shape descriptor, obtained from a PCA of the atlas velocity fields, we are able to separate the OD size variability among healthy patients from the pathological variability. Therefore, the ASD is able to characterize abnormalities from only one fundus image. We have shown that the ASD is able to distinguish normality as well as abnormalities including glaucoma. Many components of the ASD are uncorrelated with CDR measures, which confirm that all the available geometric information is not being exploited when using only the CDR. Additionally, the CDR is specific to describing glaucoma, whereas the ASD can highlight any significant deviation from the average mode and can reveal more signs than the CDR.

**Fig. 20:**
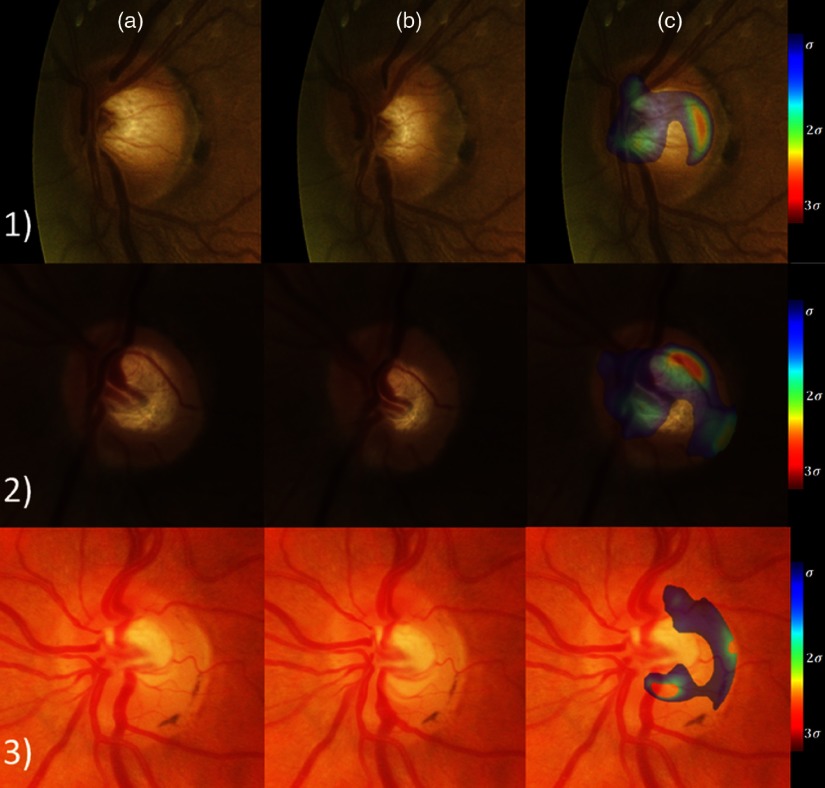
Three pathological ODs with column (a) the original cropped OD; column (b) the OD with the velocity field toward the average model applied; and column (c) the local deviation map superimposed onto the original cropped OD. The local abnormalities lie on the temporal side of the cup and also on the vessels on the nasal side (row 1), on the superior side of the cup and on the nasal rejection of the inferior vessels (row 2), and on the inferior side of the cup (row 3).

One big advantage of the atlas approach is the easiness to interpret results and what have been learned in the atlas. We have already seen that each element of the ASD has a geometric meaning and that we can visualize the modes as a geometric deformation and therefore explain on which deformation mode a pathological OD is outside the healthy variability. Furthermore, the local deviation map $d_{\mathrm{new}}$ is very useful to track the local deformations that are outside the atlas variability (see Fig. 20). The ASD is able to assist the clinicians in the assessment of pathological ODs and supplement the use of clinical measures, such as the CDR. The local deviation map highlights the local deformation and can assist the clinicians to detect these early local deformations.

To further assist the clinicians, the statistical atlas will help the clinicians in early glaucoma detection and stratification with the computation of a new glaucoma likelihood score calculated from the ASD. More generally, this statistical atlas will help to study the correlation of the ASD components with clinical assessment in pathologic cases. We will be able to analyze and quantify the morphological variability of OD region in healthy populations and better understand ethnic differences. As well, we will be able to study the asymmetry of the OD region abnormalities between right and left eyes.

In this work, the statistical atlas is constructed with a healthy OD region to model the variability of a healthy population regardless of other demographic data. With added information such as age, we would be able to construct a longitudinal statistical atlas to analyze the variability due to aging and those related to a pathology. Furthermore, the atlas construction framework could be extended to characterize 3-D deformations with optical coherence tomography exams of the OD. The 3-D shape of the OD carries useful information and the variability could be studied using the same framework by adapting the registration procedure to 3-D velocity fields.

However, the number of fundus images chosen to construct the atlas is currently limited. We chose to validate each segmentation by an ophthalmologist so that the results of the study are not biased by any segmentation errors. The similarity of the ASD components between the atlas population and the other 129 healthy population, however, indicates that the current atlas represents well an unknown healthy population. In addition, our method for constructing the average model is incremental so we can easily add new ODs to the average model. An easy way is to begin with the current average model as the representation and iterate toward the new average model. So, adding new patients to the atlas is fast, which is a real advantage of an atlas approach.

In this study, only one ophthalmologist has validated the segmentation. Thus, the interexpert variability in OD region segmentation should be studied. For future improvements, we could construct the atlas from the average segmentation of multiple experts when available.

As for now, the proposed method is semiautomatic. Full automatic cup segmentation could be achieved with the recent development of deep learning techniques. However, lack of annotated data requires realistic data augmentation, especially for medical images. Our proposed atlas would be able to procure realistic deformation of the OD region and therefore improve automatic segmentation of the OD region.

Ongoing work focus on gathering more pathological data and then proposing a method to construct specific scores derived from the ASD, specialized to detect different pathologies.
